# Editorial: Advancing the understanding of key events in the intracellular parasitism by apicomplexan parasites

**DOI:** 10.3389/fcimb.2023.1288638

**Published:** 2023-09-20

**Authors:** Kamalakannan Vijayan

**Affiliations:** School of Biology, Indian Institute of Science Education and Research, Thiruvananthapuram, Kerala, India

**Keywords:** host-parasite interactions, apicomplexan, actin dynamics, nutrient trafficking, organelle biogenesis

Apicomplexan parasites, causing diseases like malaria and toxoplasmosis, have intricate lifecycles with asexual and sexual stages adapted to different hosts. Though these parasites are confined within the protective parasitophorous vacuoles (PVs) following infiltration, they influence the host cell function by establishing contact at the PVM-host interface. This intricate interplay defines apicomplexan survival and pathogenicity within hosts.

In this editorial, we present four distinct research articles that will expand our knowledge of the comprehensive interactions between host and apicomplexan parasites.

The distinctive arrangement of organelles at the apical complex which facilitates invasion is what bestowed the name Apicomplexa. In this Research Topic, Gubbels et al., highlight the multifunctional nature of the basal complex (BC) in *Toxoplasma gondii*, an often-overshadowed structure compared to the apical complex. Few studies have highlighted a diverse range of functions associated with BC such as cell division, cytoplasmic bridge formation, intravacuolar network (IVN) assembly, etc., but the molecular underpinnings that regulate these processes are not clear. Gubbels et al., demonstrate how the BC undergoes dynamic structural changes during the discrete developmental stages throughout the lytic cycle. In addition, Gubbels et al., have made efforts to comprehensively profile the structure-function relationship of several BC resident proteins. This article amalgamates these cumulative insights with new data to present a comprehensive overview of *T. gondii’s* BC.

The dearth of standardized methods to evaluate suppression of sporozoite infection hinders the development of new interventional therapies against Cryptosporidiosis. Ogbuigwe et al., have demonstrated that a naturally occurring auto-fluorescent signal, Sig M, detected in *Cryptosporidium*-infected host cells without any fluorescent labeling strategies could be exploited for a wide variety of applications such as assessing the efficacy of antibodies/small molecules in a high-throughput scale. In addition, the ability to sort the live infected cells in an antibody/label-free manner facilitates addressing several basic biology questions through, transcriptomics and genome-wide association studies specifically in the infected population. This approach can be easily adapted for the limited quantities of samples that are typically available from clinical investigations.

*Eimeria* parasites, the causative agents of coccidiosis, are a leading cause of economically significant disease of livestock. *Eimeria* factors contributing to the invasion are well studied but little is known about the structure and functional alterations in *Eimeria* that initiate commitment towards intracellular development. Using imaging-based morphometric analysis, Burrell et al., have profiled the early alterations in sporozoites post-invasion and identified structural re-organization of refractile bodies (RB), a membrane-less organelle, following the invasion. Specifically, Burrell et al. have characterized the dynamics of the actin-dependent merger of anterior and posterior RBs following the host cell invasion and its implications in commitment towards intracellular asexual replication.

Neosporosis is another apicomplexan manifested cattle disease that results in a huge economic burden. Apicomplexans are well known to utilize actin-based mechanisms for the invasion process. *N. caninum* possesses atypical and versatile actin-binding proteins compared to higher eukaryotes. In this Research Topic, Baroni et al. have documented that the regulatory components of actin dynamics in *N. caninum* can be modulated by oxidation. This study raises an interesting question of whether the oxidative environment of the host during invasion favors the parasite actin dynamics and thereby facilitates entry by dysregulating actin-depolymerization factors.

Collectively these articles showcase the ongoing efforts and contribute to our understanding of key events in the intracellular parasitism by apicomplexan parasites.

## Author contributions

KV: Writing – original draft, Writing – review & editing.

